# Quantitative assessment of the diagnostic role of *APC* promoter methylation in non-small cell lung cancer

**DOI:** 10.1186/1868-7083-6-5

**Published:** 2014-03-24

**Authors:** Shicheng Guo, Lixing Tan, Weilin Pu, Junjie Wu, Kuan Xu, Jinhui Wu, Qiang Li, Yanyun Ma, Jibin Xu, Li Jin, Jiucun Wang

**Affiliations:** 1 Ministry of Education Key Laboratory of Contemporary Anthropology and State Key Laboratory of Genetic Engineering, School of Life Sciences, Fudan University, Shanghai 200433, China; 2 Department of Pneumology, Changhai Hospital of Shanghai, Second Military Medical University, Shanghai 200433, China; 3 Department of Head and Neck Surgery, Cancer Hospital, Fudan University, Shanghai 200032, China; 4 Department of General Surgery, University of Qingdao Affiliated Hospital of Medical College, Qingdao University, 1677 Wutaishan Street, Qingdao City 266071, China; 5 Department of Cardiothoracic Surgery, Changhai Hospital of Shanghai, Second Military Medical University, Shanghai, China

**Keywords:** *APC*, DNA methylation, Diagnosis, Meta-analysis, TCGA, NSCLC, Biomarker

## Abstract

**Background:**

Adenomatous polyposis coli (*APC*) has been reported to be a candidate tumor suppressor in many cancers. However, the diagnostic role of *APC* promoter methylation in non-small cell lung cancer (NSCLC) remains unclear. We systematically integrated published articles and DNA methylation microarray data to investigate the diagnostic performance of the *APC* methylation test for NSCLC. Two thousand two hundred and fifty-nine NSCLC tumor samples and 1,039 controls were collected from 17 published studies and TCGA NSCLC data. The association between *APC* promoter methylation and NSCLC was evaluated in a meta-analysis. An independent DNA methylation microarray dataset from TCGA project, in which five CpG sites located in the promoter region of *APC* were involved, was used to validate the results of the meta-analysis.

**Results:**

A significant association was observed between *APC* promoter hypermethylation and NSCLC, with an aggregated odds ratio (OR) of 3.79 (95% CI: 2.22 to 6.45) in a random effects model. Pooled sensitivity and specificity were 0.548 (95% CI: 0.42 to 0.67, *P* < 0.0001) and 0.776 (95% CI: 0.62 to 0.88, *P* < 0.0001), respectively. Each of the five CpG sites was much better in prediction (area under the curve, AUC: 0.71 to 0.73) in lung adenocarcinoma (Ad) than in lung squamous cell carcinoma (Sc) (AUC: 0.45 to 0.61). The AUCs of the logistic prediction model based on these five CpGs were 0.73 and 0.60 for Ad and Sc, respectively. Integrated analysis indicated that CpG site location, heterogeneous or autogenous controls, and the proportion of adenocarcinoma in samples were the most significant heterogeneity sources.

**Conclusions:**

The methylation status of *APC* promoter was strongly associated with NSCLC, especially adenocarcinoma. The *APC* methylation test could be applied in the clinical diagnosis of lung adenocarcinoma.

## Background

Non-small cell lung cancer (NSCLC), including adenocarcinoma (Ad) and squamous cell carcinoma (Sc), is the leading cause of cancer death in both men and women in the United States [1]. Over 159,480 Americans die of this disease every year in the US [1]. The five-year relative survival rate varies markedly depending on the stage at diagnosis, from 49% to 16% to 2% for patients with local, regional, and distant stage disease, respectively (SEER Cancer Statistics Review 1975 to 2002). However, the bottleneck in improving survival is early detection [2]. As an important mechanism for tumor suppressor gene inactivation in cancer, DNA hypermethylation could yield powerful biomarkers for early detection of lung cancer, owning incomparable advantages over other traditional markers due to its stable chemical property, detection ability in remote patient media, quantitative signal, convenient low cost in detection, and so on [3]. Several revolutionary steps have been made to promote application of methylation biomarkers in cancer screening [4,5]. Therefore, we believe that DNA methylation could become a powerful tool for lung cancer diagnosis.

The *APC* gene encodes a tumor suppressor protein that acts as an antagonist of the Wnt signaling pathway, and it also participates in cell migration and adhesion, transcriptional activation, and apoptosis [6]. Meanwhile, defects in the *APC* gene cause familial adenomatous polyposis (FAP), an autosomal dominant pre-malignant disease that usually progresses to malignancy, suggesting that *APC* could be a potential predictor for cancer initiation or development. Researchers have reported that promoter methylation, which inhibits *APC* gene expression, is mediated by changes of chromatin conformation and aberrant binding of CCAAT-box binding transcription factors [7].

Like P16INK4A [8], the relationship between hypermethylation of *APC* with cancers has also been extensively estimated [9] and *APC* promoter hypermethylation in NSCLC has been reported as an effective biomarker for diagnosis [10,11]. However, the results appear dramatically different among different research studies, and this may be caused by the difference in gender proportion, age distribution, racial source, certain other epidemiological characteristics in samples, detection methods, and so on. In addition, there has not yet been any quantitative assessment of the relationship between hypermethylation in the promoter region of the *APC* gene and NSCLC.

In this article, we conducted a meta-analysis of the sensitivity and specificity of *APC* methylation on NSCLC diagnosis. The factors which lend heterogeneity to the sensitivity and specificity were identified with meta-regression. We also found that The Cancer Genome Atlas project (TCGA) had collected hundreds of whole genome DNA methylation microarray datasets of NSCLC samples which included comprehensive clinical and demographic information, providing an additional resource that may be without publication bias. In our work, we innovatively integrated these TCGA data (Additional file 1: Table S1) and the data from published articles to evaluate the diagnostic ability of the *APC* methylation test in NSCLC. Therefore, an integrated analysis of all these existing data was conducted to come to unbiased conclusions on the relationship between *APC* methylation and NSCLC.

## Results

### Study characteristics

The electronic search strategy identified 506 potentially relevant articles (PubMed, 315; Scopus, 112; Cochrane Library, 3; OVID Medline, 53; TMC ProSearch, 23), which were further screened for inclusion on the basis of their titles, abstracts, full texts, or a combination of these terms. The electronic search was supplemented from reference lists of relevant articles including reviews. Finally, 17 studies with data on the relationship between *APC* gene promoter methylation and NSCLC were pooled for analysis (Table 1) [10,12-27]. All these articles were written in English. In total, 1,338 lung cancer tissues/serum and 913 normal counterpart tissues/serum were collected. The age of the subjects in the 17 studies ranged from 25 to 86 years, with mean or median ranging from 53 to 67 years. As for the study aim, 13 articles were especially aiming at diagnosis, while the others were for prognosis, survival research, and so on. Among 17 studies, the proportions of stage I samples differed from 32.1 to 100%, and the percentage of male individuals in the NSCLC samples has a range of 53 to 81%. For the experimental methods to explore *APC* promoter methylation status, 7 of 17 inclusions used methylation-specific polymerase chain reaction (MSP), while others used quantitative MSP (qMSP, such as Methylight, Prosequencing, and so on). Two kinds of methylation detection primers or probes were found to be utilized for most of the 17 studies. The information of the two sets of primers (set I: chr5:112073421-112073518, seven studies; and set II: chr5:112101379-112101452, seven studies) is listed in Additional file 1: Table S2. Although no CpG sites from the methylation microarrays were found located in the above primers, cg20311501 is covered by the replication region of set II primers.

**Table 1:** Characteristics of eligible studies considered in the report

**Author**	**Sample type**	**Age**^**a**^	**Stages I%**	**Gender (M/F)**	**Patients (M+/M-)**	**Control (M+/M-)**	**Methods**	**Aim**	**Multiple target**	**Ad2Sc**	**Control design**	**Reference**
Zhang *et al*. [27]^b^	Tissue	59	32.05	29/39	44/34	10/68	MSP	Diagnose	Yes	0.83	hom	[27]
Wang *et al*. [25]	Tissue	-	-	17/28	19/9	1/11	qMSP	Diagnose	Yes	2.14	heter	[25]
Jin *et al*. [15]	Tissue	66.7	-	17/24	27/45	22/41	qMSP	Non-diagnose	Yes	1.87	heter	[15]
Feng *et al*. [14]	Tissue	64.3	42.86	26/49	26/23	21/28	qMSP	Diagnose	Yes	1.43	hom	[14]
Brabender *et al*. [13]	Tissue	63.3	49.45	69/91	86/5	80/11	qMSP	Non-diagnose	Single	0.77	hom	[13]
Virmani *et al*. [24]	Tissue	-	-	-	22/26	0/18	MSP	Diagnose	Yes	NA	heter	[24]
Yanagawa *et al*. [26]	Tissue	67.3	66.67	18/25	28/47	36/39	MSP	Diagnose	Yes	1.48	hom	[26]
Topaloglu *et al*. [22]	Tissue	-	54.84	-	17/14	5/17	qMSP	Diagnose	Yes	3.00	heter	[22]
Kim *et al*. [16]	Tissue	63	56.57	64/79	48/41	33/66	MSP	Non-diagnose	Yes	0.62	hom	[16]
Vallbohmer *et al*. [23]	Tissue	63	49.45	69/91	86/5	80/3	PCR	Non-diagnose	Yes	0.77	hom	[23]
Lin *et al*. [17]	Tissue	61.1	100.00	20/31	49/75	2/24	MSP	Diagnose	Yes	1.84	heter	[17]
Shivapurkar *et al*. [20]	Tissue	-	-	-	35/5	23/17	qMSP	Diagnose	Yes	1.22	heter	[20]
Suzuki *et al*. [21]	Tissue	64	34.00	33/49	53/97	3/57	MSP	Non-diagnose	Yes	NA	heter	[21]
Zhang *et al*. [27]^b^	Serum	-	-	-	54/56	5/45	MSP	Diagnose	Yes	NA	heter	[27]
Pan *et al*. [18]	Serum	53	-	17/26	40/38	0/31	qMSP	Diagnose	Single	NA	heter	[18]
Begum *et al*. [12]	Serum	65	-	10/19	12/64	3/27	qMSP	Diagnose	Yes	NA	heter	[12]
Rykova *et al*. [19]	Serum	NA	-	-	3/6	0/16	MSP	Diagnose	Yes	NA	heter	[19]
Usadel *et al*. [10]	Serum	64.2	-	-	42/47	0/50	qMSP	Diagnose	Single	NA	heter	[10]

^a^mean or median age from articles; ^b^with two records since there are Tissue and serum data simultaneously in this article. M + and M- means methylation positive and methylation negative, respectively.

Abbreviations*: Ad2Sc*, the ratio of adenocarcinoma to squamous cell carcinoma; *hom*, homogeneous control; *heter*, heterogeneous control. *MSP*, qualitative methylation detection method; *qMSP*, quantitative detection method; Sample type represents the samples analyzed.

### Meta-analysis, subgroup analysis and meta-regression

**Figure 1:**
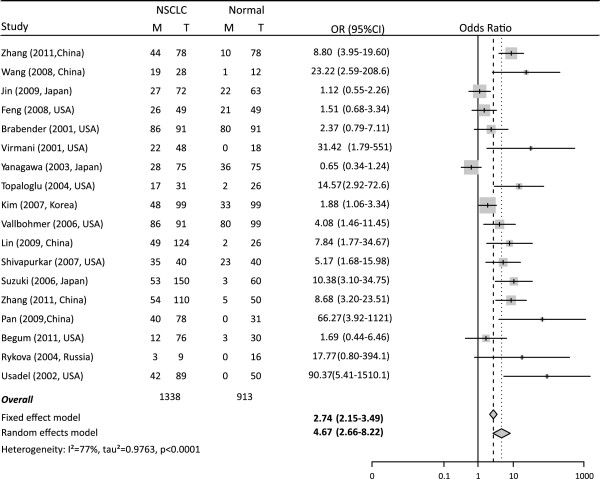
**Combined estimates for the association between *APC *promoter hypermethylation and non-small cell lung cancer (NSCLC) with forest plot.** Author, year, country of the studies and methylated (M) and total number of the sample (T) in case and control, combined odds ratio (OR) with 95% confidence region were labeled in the left column of the figure. The DerSimonian-Laird estimator and Mantel-Haenszel method were selected to conduct combination estimation for the random effects model and fixed effects model, respectively.

The ORs for *APC* methylation in cancer tissues compared with that in normal controls were 4.67 (95% CI: 2.66 to 8.22, z = 5.35, *P* < 0.0001) in random effects model pooled, and 2.74 (95% CI: 1.99 to 3.23, z = 8.10, *P* < 0.0001) in fixed effects model, demonstrating a statistically significant increasing in likelihood of methylation in lung cancer tissues comparing to controls (Figure 1).

**Figure 2:**
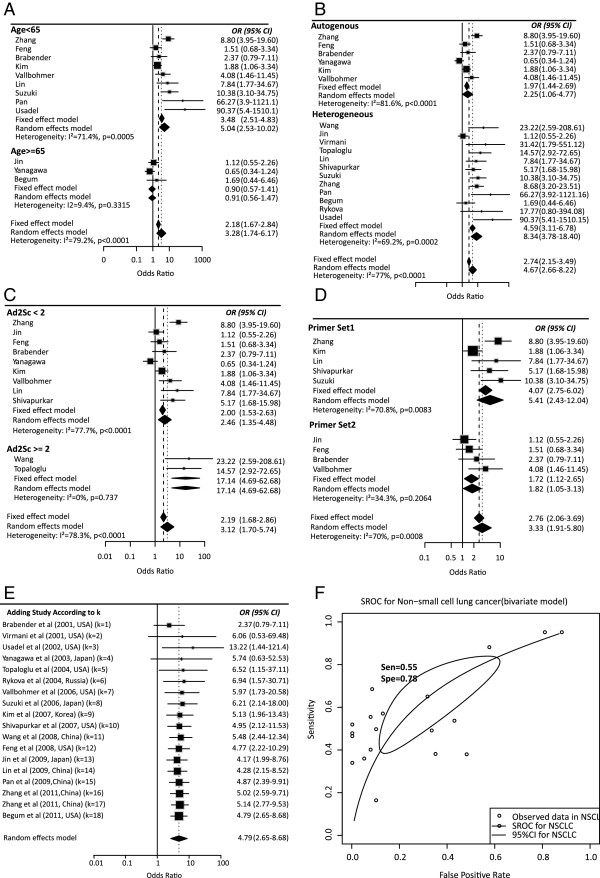
**Subgroup meta-analysis, cumulative analysis and summary receiver operating characteristics (SROC) estimation for the relationship between APC promoter hypermethylation and non-small cell lung cancer (NSCLC). (A**-**D)** Subgroup meta-analysis based on age, control type, percentage of adenocarcinoma in total samples and primer set, respectively. **(E)** Cumulative meta-analysis of studies ordered chronologically by publication year with random effects model. **(F)** SROC of *APC* methylation test in NSCLC.

Subgroup analyses were conducted for different subtypes, which included sample types (tissue or serum), counterpart categories (autogenous or heterogeneous), proportion of stage I, aim of the study (for diagnosis or non-diagnosis), ratio of adenocarcinoma to squamous (Ad2Sc), primer categories (sets I and II) (Additional file 1: Table S2) and other possible confounding factors (Table 2). Significant differences were found between the ORs of the younger (5.03, 95% CI: 2.53 to 10.0) and older (0.91, 95% CI: 0.57 to 1.41) subgroup (*P* < 0.0001) (Figure 2A). The group with the high proportion of adenocarcinoma had a significantly bigger OR than that of the low subgroup (*P* = 0.0077) which suggested that *APC* methylation might have subtype specificity in NSCLC (Figure 2C). Significant difference was also found between primer sets I and II (*P* = 0.0137), which supported primers as being one of the most important heterogeneity sources in the *APC* methylation test (Figure 2D). Both tissue and serum groups showed significant association between *APC* methylation and NSCLC (OR = 3.72, 11.54, respectively) which suggested that *APC* methylation can be taken as a potential biomarker for NSCLC diagnosis using either tissue or serum samples. In addition, significant differences were found between the ORs of heterogeneous (OR_h_ = 8.33, 95% CI: 3.77 to 18.39) and autogenous (OR_a_ = 2.25, 95% CI: 1.06 to 4.77) subgroups (*P* = 0.0187) (Figure 2B). One possible reason might be the impure composition of the adjacent normal specimens which might have been slightly contaminated by cancer cells, or it have been transformed to precancerous status, while normal serum samples generally came from healthy individuals. The subgroup of high Ad2Sc had a larger OR than that of low Ad2Sc (Table 2), indicating that methylation of *APC* might have occurred or functioned at the early stage of tumorigenesis, which had been found for endometrial cancer [28]. The OR in studies aiming at diagnosis (OR = 6.79) is more than 2.6 times the OR in the non-diagnosis group (OR = 2.59), which might be caused by an unbalanced distribution in the proportion of early stage samples (*P* = 0.0218, Wilcoxon rank-sum test). No significant difference was found between subgroups of MSP and qMSP (*P* = 0.77), which suggested both of the methods were equivalent in methylation detection (Table 2) and the result was consistent with Wu’s conclusion [29].

**Table 2:** Subgroup analysis for the main potential confounding factors with random effects model

**Subgroup**	**Number of study**	**OR**	**95% CI**	**Q**	**I**^**2**^	***P*-value**
Overall	18	4.67	2.65 to 8.21	73.99	77.00%	
Age ≤ 65	9	5.03	2.53 to 10.0	27.96	71.40%	
Age > 65	3	0.91	0.57 to 1.41	2.21	9.400%	**< 0.0001**
Stage I > 49.5%	5	4.11	1.90 to 8.91	12.76	68.60%	
Stage I ≤ 49.5%	4	2.81	0.87 to 9.09	19.42	84.60%	0.5944
M2F ≤ 69%	6	5.98	2.04 to 17.53	16.66	70.00%	
M2F > 69%	6	2.13	0.99 to 4.55	29.05	82.80%	0.1246
MSP	8	5.16	2.01 to 13.26	44.61	84.30%	
qMSP	10	4.32	2.08 to 8.94	29.28	69.30%	0.7685
Diagnose	13	6.79	2.99 to 15.44	59.54	79.80%	
Non-diagnose	5	2.59	1.33 to 5.05	11.56	65.40%	0.0745
Multiple targets	15	4.08	2.28 to 7.34	62.99	77.80%	
Single target	3	18.72	1.23 to 283	9.03	77.80%	0.2836
Heterogeneous	12	8.33	3.77 to 18.39	35.71	69.20%	
Autogenous	6	2.25	1.06 to 4.77	27.19	81.60%	**0.0187**
Serum	5	11.54	2.87 to 46.40	10.4	61.50%	
Tissue	13	3.72	2.03 to 6.78	55.18	78.30%	0.14
Ad2Sc < 2	9	2.46	1.35 to 4.48	35.79	77.00%	
Ad2Sc > = 2	2	17.1	4.68 to 62.7	0.11	0.000%	**0.0077**
Primer set I	5	5.41	2.43 to 12.04	13.71	70.80%	
Primer set II	4	1.82	1.05 to 3.13	4.57	34.30%	**0.0137**^**a**^

Bold *P*-values lower than 0.05 indicate significant differences between groups (random effects model, d.f. = 1).

^a^The serum groups and the studies with less than 50 samples were removed.

Meta-regression revealed that heterogeneity exists among 17 studies (I^2^ = 79.2%, Q = 52.78, *P* < 0.0001) (Figure 1), whereas age and primer sets were the major source of heterogeneity. The trend in ORs was inversely correlated with age (beta = -0.3, *P* = 2.0 × 10^-5^), and age counted for 83.8% total variances. This result is consistent with the subgroup analysis, in which the OR of the older group (OR = 2.24) was smaller than the younger group (OR = 4.65). The primer set is also an important heterogeneity source (*P* = 0.05), explaining about 68% of overall heterogeneity. Other factors such as sample type, proportion of males, proportion of stage I and detection methods fail to explain the heterogeneity counting for type I error at level of 0.05 (Table 3).

**Table 3:** Meta-regression analysis for the main potential interference factors with random-effects model

**Subgroup**	**Coefficient (95% CI)**	***P*-value**	**τ**^**2**^
Sample type	-1.03 (-2.4, 0.34)	0.14	0.90
Age	-0.3 (-0.44, -0.16)	**2.0 × 10**^**-5**^	0.18
Proportion of stage I	-0.01 (-0.05, 0.03)	0.608	0.79
Ratio of male to female	-0.69 (-8.1, 6.71)	0.855	0.98
Detection methods	-0.09 (-1.28, 1.1)	0.88	1.11
Study aim	-0.82 (-2.05, 0.41)	0.19	1.07
Single/Multiple targets	1.05 (-0.71, 2.81)	0.243	1.01
Hetero/Autogenous control	-1.25 (-2.35, -0.15)	0.026	0.89
Ad2Sc	0.44 (-0.56 , 1.44 )	0.387	0.89
Primer set	-1.02 (-1.02, -2.02)	**0.05**	0.35

Bold *P*-values lower than 0.05 indicate the item would be a significant heterogeneity.

### Summary receiver operating characteristic curve for diagnostic capacity of *APC* methylation

Pooled sensitivity and specificity were 0.548 (95% CI: 0.42 to 0.67, *P* < 0.0001) and 0.78 (95% CI: 0.62 to 0.88, *P* < 0.0001) for all the studies based on the presupposition of the fixed effects model. The sensitivity of the tissue group was higher than that of the serum group, 0.61 (0.45 to 0.75) versus 0.396 (0.26 to 0.56), while the specificity of the serum group was higher than that of the tissue group, 0.92 (0.86 to 0.96) versus 0.68 (0.49 to 0.83), which suggested the advantage of this biomarker for its higher diagnostic ability using remote non-invasive media.

Although sensitivity and specificity were two of most important features of a diagnostic test, in some occasions, pooling sensitivity or specificity could be misleading as mentioned in the Methods section. Therefore, we constructed the summery receiver operating characteristic (SROC) curve to depict the stability and accuracy of the methylation test’s diagnostic ability. The area under the curve (AUC) of the SROC was 0.64, suggesting a fair ability for NSCLC diagnosis (Figure 2F). Meanwhile, the AUC of the SROC for the serum and the tissue group was 0.67 and 0.64 respectively, showing slightly different performances for the *APC* methylation test in serum and tissue samples.

### Bias analysis and robust estimation of pooled OR

A funnel plot of methylation status of lung cancer tissue versus normal tissue showed significant publication bias (Egger test, z = 4.3, *P* < 0 .0001) and eight studies exceeded the 95% confidence limits (Additional file 2: Figure S1). In order to eliminate the effect of publication bias, trim and fill analysis was performed with the random effects model. The adjusted pooled OR were 2.50 (95% CI: 1.43 to 4.38, *P* = 0.0013) in the random effects model and 2.19 (95% CI: 1.74 to 2.77, *P* < 0.0001) in the fixed effects model. Both results demonstrate a significantly positive association between *APC* methylation and NSCLC (Additional file 2: Figure S2).

In sensitivity analysis to determine the effect of omitting a single study on the overall effect, the overall ORs were between 4.3 (95% CI: 2.46 to 7.52) and 5.27 (95% CI: 2.92 to 9.53) in the random effects method, which suggested that combined OR was consistent and reliable (Additional file 2: Figure S3).

A cumulative meta-analysis at the time of the published literature was also conducted, and we found the OR was tending to be stable (Figure 2E). The stable result indicates our meta-analysis might be more credible when more incoming researches are added.

Using similar methodology, the influence on meta-regression was determined by omitting one study each time to explore heterogeneity sources. The sample type of tissue or serum would be one of the heterogeneity sources (*P* < 0.026) when Begum *et al*. ([12], US) were removed from the meta studies; likewise, the proportion of stage I and aim of the study would become the heterogeneity source when Lin *et al*. ([17], China), Zhang *et al*. ([27], China) or Yanagawa *et al*. ([26], Japan) was removed (*P*-values were 0.0046, 0.029 and 0.039 respectively). This analysis suggested the above factors should be considered in a future case-control association study.

### Validation by independent TCGA lung cancer dataset

In order to make independent validation of the above results, we collected the data of the methylation status of six CpG sites located in the promoter region of the *APC* gene from the lung cancer samples of TCGA project (Additional file 1: Table S1). Pairwise methylation Pearson correlation analysis showed that the methylation status was highly correlated among these CpG sites (R^2^ > 0.90 for all) except cg01240931 (R^2^ < 0.45 for all), which suggested that cg01240931 was out of the ‘methylation block’ composed of the other five CpG sites. Meanwhile, cg01240931 was hypermethylated in both the cancer and normal specimens. Therefore, this CpG site was excluded in the following analysis.

The clinical characteristics of the NSCLC samples were extracted from TCGA project. There is no significant difference in age or gender between the cases and controls (data not shown). The methylation percentages of cg15020645, cg16970232, cg20311501, cg21634602 and cg24332422 were dramatically different between the two groups, especially in adenocarcinoma. The methylation of all these five CpG sites were significantly different between Ad and its counterparts according to the *t*-test after FDR adjustment (*P* < 10^-17^), whereas only two CpG sites (cg16970232, cg20311501) were significantly different between Sc and its counterparts (*P* = 1.6 × 10^-6^ and 3.9 × 10^-3^) (Table 4). In addition, logistic regression analysis also supported the above results: the ORs in Ad were from 23.3 to 1.2 × 10^3^, while those were from 0.15 to 7.54 in Sc (Table 4). The AUCs of the five CpG methylation tests were calculated to assess their prediction ability. As shown in Table 4, each of the CpG sites in Ad was a much better predictor (AUC: 0.71 to 0.73) than that in Sc (AUC: 0.45 to 0.61). The AUCs of the logistic prediction model based on all the five CpG sites were 0.73 for Ad and 0.60 for Sc. All the results above indicate that the *APC* methylation test would have better performance in adenocarcinoma than that in Sc, and therefore, the variation in the proportions of Ad and Sc in the samples might affect the association between *APC* methylation and NSCLC. Generally, 25 to 30% of lung cancers were Sc while 40% were Ad. Thus, we resampled the Ad and Sc from TCGA data to simulate the effect of the different ratios of Ad versus Sc (Ad2Sc) at 2:1, 4:3, 3:4 and 1:2 on the OR of *APC* methylation for NSCLC. The ORs dramatically varied within group and between groups of the five CpGs by 10,000 times of resampling simulations (Additional file 1: Table S3). As expected, cg16970232 and cg20311501, the two significant sites in both Ad and Sc, were consistently significant risk factors for NSCLC, while the other three CpGs lost association with NSCLC in certain vignettes (Additional file 1: Table S3). Moreover, ORs from logistic regression based on heterogeneous samples were significantly greater than those of the autogenous samples in the condition of Ad2Sc of 4:3, which was concordant with the above subgroup meta-analysis (Additional file 1: Table S4). Logistic interaction analysis among age and gender with NSCLC did not show statistically significant interaction between *APC* methylation and these covariates in the risk of NSCLC (Additional file 1: Table S5).

**Table 4:** **Differential *APC *** **methylation, odds ratio, area under the curve (AUC) between adenocarcinoma, squamous cell carcinoma and their counterparts**

	**Adenocarcinoma**								**Squamous Cell Carcinoma**							
**CpG site**	**MCaM (N = 535)**	**MCoM (N = 56)**	***P*-value**^**a**^	**FDR**^**a**^	**OR**^**b**^	***P*-value**^**b**^	**95% CI**^**b**^	**AUC**^**b**^	**MCaM (N = 386)**	**MCoM (N = 70)**	***P*-value**^**a**^	**FDR**^**a**^	**OR**^**b**^	***P*-value**^**b**^	**95% CI**^**b**^	**AUC**^**†**^
cg15020645	0.26(40.7%)	0.13(0%)	3.5 × 10^-32^	**1.0 × 10**^**-31**^	190.6	**7.7 × 10**^**-6**^	22.65 to 2,321	0.72	0.13(14.77%)	0.11(0%)	0.087466	0.131199	3.16	0.406	0.28 to 68.72	0.61
cg16970232	0.3(45.2%)	0.11(0%)	5.0 × 10^-38^	**3.0 × 10**^**-37**^	108.9	**5.1 × 10**^**-6**^	17.64 to 1,043	0.73	0.15(18.91%)	0.09(0%)	2.7 × 10^-7^	**1.6 × 10**^**-6**^	7.54	**0.035**	1.39 to 64.07	0.45
cg20311501	0.33(48.4%)	0.16(5.3%)	1.4 × 10^-22^	**2.1 × 10**^**-22**^	61.56	**4.96 × 10**^**-6**^	11.94 to 420	0.73	0.18(19.95%)	0.14(0%)	0.001955	**0.003909**	2.48	0.257	0.57 to 13.74	0.49
cg21634602	0.33(47.4%)	0.16(7.1%)	3.6 × 10^-17^	**4.3 × 10**^**-17**^	23.34	**3.6 × 10**^**-5**^	5.75 to 116.0	0.71	0.16(20.47%)	0.14(7.14%)	0.222306	0.266767	1.27	0.726	0.35 to 5.42	0.53
cg24332422	0.26(40.5%)	0.16(0%)	1.0 × 10^-26^	**2.0 × 10**^**-26**^	223.6	**2.81 × 10**^**-5**^	21.11 to 3,463	0.71	0.16(17.36%)	0.15(0%)	0.338755	0.338755	1.6	0.656	0.23 to 14.30	0.52

MCaM, MCoM represent the mean of case methylation (Beta) and mean of control methylation (Beta). Methylation levels are calculated with formula: Beta = (M/M + U).

^a^*P*-values are from *t*-test or Wilcoxon rank-sum test after false discovery rate (FDR adjustment). The *t*-test will be chose when the variable is normally distributed, or else the rank-sum test will be chose. Significant *P*-values after FDR are bolded which indicate significant different methylation between case and control.

^b^OR and its 95% CI, *P*-value and AUC from logistic regression analysis or prediction model.

## Discussion

The *APC* gene has been reported as an important tumor suppressor in colorectal cancer [30], and the aberrant of *APC* methylation had been reported in numerics for cancers, such as bladder [31], prostate [32], breast and lung cancer [24]. However, the diagnostic role of the methylation status of the *APC* gene in lung cancer lacks quantitative assessment. We therefore performed an integrated analysis to quantify the ability for the *APC* promoter methylation test in NSCLC diagnosis, and a significant association was identified between *APC* methylation and NSCLC (OR = 4.67, *P* < 0.0001). Seven imputed studies were filled when trim and fill tests were performed to eliminate the influence of publication bias on the random effects model, and the overall OR (2.49, 95% CI: 1.18 to 5.26) was still significant, although it was slightly smaller than that in the crude meta-analysis (4.67, 95% CI: 2.66 to 8.22), indicating the existence of a strong association between *APC* promoter methylation and lung cancer. The pooled sensitivity, specificity and AUC of the *APC* methylation test in the present meta-analysis were 0.548, 0.78 and 0.64, respectively, which revealed that *APC* methylation status is a good biomarker in NSCLC diagnosis.

Integrated analysis showed that the age at diagnosis, autogenous or heterogeneous control, the ratio of adenocarcinoma to squamous cell carcinoma, and primer set of CpG sites were the most important heterogeneity sources, while sample type (tissue or serum), proportion of males, proportion of stage I, and detection methods could not explain the heterogeneity.

Age was one of the most important heterogeneity sources from meta-regression analysis (beta = -0.3, *P* = 2.0 × 10^-5^), meanwhile, the OR in the younger subgroup (OR = 4.65) was greater than that in the older subgroup (OR = 2.24). However, TCGA NSCLC datasets demonstrated different results. Furthermore, neither Ad nor Sc data supported age affecting the OR of the *APC* methylation to the risk of NSCLC in the logistic regression model (*P* > 0.05). Briefly, much more evidence should be collected before making a final decision.

As to the contribution of Ad2Sc, both subgroup analysis and TCGA analysis showed significantly greater OR in the high Ad2Sc than that in the low Ad2Sc group, which suggested the *APC* methylation test has better diagnostic performance for adenocarcinoma.

Since the late 1980s, various studies have shown that the same genetic/epigenetic alterations, such as DNA methylation, in the primitive tumors were also found in the circulating DNA of the patients with tumors [33-35]. Interestingly, in the present study, the OR of the serum subgroup was greater than that of the tissue group and the AUC of the *APC* methylation test for serum was greater than that for tissue in both meta- and microarray analysis, which indicated that the *APC* methylation test should be a promising serum biomarker for NSCLC diagnosis.

Meta-analysis has been widely applied in SNP-disease risk association studies because SNPs have specific genome location. Meta-analysis is also gradually starting to boom in the realm of DNA methylation. Here, the primers for methylation detection have been considered when extracting information from studies; however, they have sometimes been difficult to analyze in the subsequent subgroup or meta-regression analysis due to the great diversity of the primers used in each individual article. For example, at least three different primer sets were observed in the 17 studies we selected for meta-analysis (Additional file 1: Table S2). Moreover, in order to expatiate on the divergence of different CpG sites, we collected the methylation signals of five CpGs from the methylation 27 K and 450 K microarray datasets from TCGA project (Ad and Sc). It was found that the ORs of the five CpG sites were dramatically different (Table 4). Subgroup analysis further showed significantly different ORs in different primer sets. This reminds us that future DNA methylation detection in case-control studies should be designed more accurately and comprehensively for certain CpG sites or blocks and the location information should be clearly noted when published in order to facilitate the re-analysis of the published data.

## Conclusion

In conclusion, this integrated analysis of the pooled data provides strong evidence that the methylation status of the *APC* promoter is strongly associated with NSCLC, especially for adenocarcinoma. Therefore, the *APC* methylation test could be a promising diagnostic biomarker which could be applied in the clinical diagnosis of lung adenocarcinoma with remote non-invasive media detection.

## Methods

### Search strategy, selection of studies and data extraction

This pooled study involved searching a range of computerized databases, including PubMed, Cochrane Library, OVID Medline and TMC ProSearch for articles published in English or Chinese by September 2013. The study used a subject and text word strategy with (*APC* OR *BTPS2* OR *DP2* OR *DP2.5* OR *DP3* OR *PPP1R461*) AND (Lung OR NSCLC) AND (cancer OR neoplasm)) as the primary search terms. Wildcard character of star, dollar or some other truncations were applied according to the rules of the databases to allow effective article collection.

Two independent reviewers (Guo, Tan) screened the titles and abstracts derived from the literature search to identify relevant studies. The following types of studies were excluded: animal experiments, case reports, reviews or meta-analyses and studies of non-case-control studies or studies with insufficient data or those proving inaccessible after making contact with the authors. The remaining articles were further examined to see if they met the inclusion criteria: 1) the patients had to be diagnosed with NSCLC (Ad and Sc), 2) the studies had to contain *APC* gene promoter methylation data from tissue, blood or serum, 3) the studies had to be case-control studies which included tissue-tissue, blood-blood or serum-serum in case and controls respectively. The reference sections of all retrieved articles were searched to identify further relevant articles. Potentially relevant papers were obtained and the full text articles were screened for inclusion by two independent reviewers (Guo, Tan). Disagreements were resolved by discussion with KX, JJW, and JHW. Included studies were summarized in data extraction forms. Authors were contacted when relevant data were missing. The name of the first author, year of publication, sample size, age (mean or median), gender proportion (male/female, M2F), the proportion of TNM stage I samples (proportion of early stage of NSCLC samples), publication aim (for diagnosis or not), analyzing multiple genes or not (one or more genes detected simultaneously in studies design), control type (autogenous or heterogeneous counterpart) and methylation status of the *APC* promoter in human NSCLC and normal or control tissues were extracted.

### Meta-analysis and SROC analysis

Data were analyzed and visualized mainly using R Software (R version 2.15.3) including meta, metefor and mada packages. The strength of association was expressed as pooled odds ratio (OR) with corresponding 95% confidence intervals (95% CI). Data were extracted from the original studies and recalculated if necessary. Heterogeneity was tested using the I^2^ statistic with values over 50% and Chi-squared test with *P* ≤ 0.1 indicating strong heterogeneity between the studies [36]. Tau-squared (τ^2^) was used to determine how much heterogeneity was explained by subgroup differences. The data were pooled using the DerSimonian and Laird random effects model (I^2^ > 50%, *P* ≤ 0.1) or fixed effects model (I^2^ < 50%) according to heterogeneity statistic I^2^[37]. A two-sided *P* ≤ 0.05 was considered significant without special annotation. Random effects meta-regression, was employed to determine how much of the heterogeneity (between-study variance) is explained by the explanatory variables when the heterogeneity was significant [38]. Nine variables were analyzed in meta-regression, including control types (autogenous and heterogeneous), gender proportion, proportion of TNM stage I samples, mean or median age (> 65 or ≤ 65), single or multiple target detection, sample types (serum or tissue), methylation detection methods (MSP, qMSP), study designs (diagnosis or non-diagnosis) and primer sets. Sensitivity analyses were performed to assess the contributions of single studies to the final results with the abandonment of one article each time. Publication bias was analyzed by funnel plot with mixed-effects version of the Egger test. If bias was suspected, the conventional meta-trim method was used to re-estimate the effect size.

Compared with traditional SNP association studies, methylation-associated research might be involved with different methylation-definition thresholds. In these cases, traditional weighted averages (pooled sensitivity and specificity) would not reflect the overall accuracy of the test, because the extremes of threshold criteria could skew the distribution, known as the threshold effect [39]. Thus, SROC analysis was applied to meta-analysis of diagnostic tests [39,40]. The SROC curve shows the performance of the diagnostic ability of *APC* methylation to NSCLC. Each study produces values for sensitivity, specificity and therefore true positive rate (TPR) and false positive rate (FPR), and the plots were placed over the TPR and FPR points to form a smooth curve. A linear regression model was selected to fit the SROC curve where sensitivity and (1-specificity) are transformed into complex logarithmic variables. The exact AUC for the SROC function was used to assess the accuracy of the test [39].

### TCGA data extraction and analysis

DNA methylation information for NSCLC, which included two sets of samples (535 Ads and 50 controls, and 385 Scs and 67 controls), was collected from TCGA project including methylation 27 K and 450 K datasets [http://cancergenome.nih.gov/]. The estimation of methylation for each CG probe was calculated with the traditional function:

$$\mathrm{beta}=\frac{\max\;\left(M,\;0\right)}{\max\;\left(M,\;0\right)+\;\max\;\left(U,\;0\right)}$$

M and U represent the mean signal intensities for about 30 replicate methylated (M) and unmethylated (U) probes on the array. The methylation signals of the 25,978 shared CpG sites by 27 K and 450 K datasets were extracted and the methylation status of each probe was defined according to the beta-value. The CpG site will be considered methylated when the beta-value is greater than the empirical threshold of 0.3 for tissue data [41]. Six CpG sites located in the promoter region of the *APC* gene (cg01240931, cg15020645, cg16970232, cg20311501, cg21634602 and cg24332422) were taken as the object of study (Additional file 1: Table S1). Adjustment for multiple testing of differential methylation was conducted with the method of Benjamini and Hochberg at the 5% FDR level.

## Abbreviations

APC: adenomatous polyposis coli; NSCLC: non-small cell lung cancer; MSP: methylation specific PCR; SROC: summary receiver operating characteristics; AUC: area under the curve; TCGA: the cancer genome atlas project; TPR: true positive rate; FPR: false positive rate.

## Competing interests

The authors declare that they have no competing interests.

## Authors’ contributions

SG and JW, LJ, JX contributed to the conception, design and final approval of the submitted version. SG, LT, KX, JW, JW, QL, YM contributed to the meta-analysis and interpretation of data, SG WP contributed to TCGA NSCLC data analysis, All authors read and approved the final manuscript.

## Supplementary Material

Additional file 1: Table S1 TCGA probe information in this study. **Table S2.** Three kinds of primers of the present 17 studies. **Table S3.** The fluctuation of odds ratio in vignettes of different proportion of Ad. **Table S4.** Odds ratio difference between heterogeneous and autogenous samples in vignettes of different proportion of Ad. **Table S5.** Interaction estimation between CpG methylation and age, gender, TNM in Ad and Sc. Click here for file

Additional file 2: Figure S1 Funnel plot to diagnosis of the publication bias. **Figure S2.** Combined estimates for the association between APC methylation and NSCLC after trim-fill treatment. **Figure S3.** Sensitivity analyses of the overall effect by omitting a single study. Click here for file
