# Supplementary material for: Quantitative assessment of the diagnostic role of APC promoter methylation in non-small cell lung cancer
Source: Clin Epigenetics. 2014 Mar 24;6(1):5. doi: 10.1186/1868-7083-6-5 (PMC3997934; doi:10.1186/1868-7083-6-5)
Supplement: Additional file 1: Table S1 — TCGA probe information in this study. Table S2. Three kinds of primers of the present 17 studies. Table S3. The fluctuation of odds ratio in vignettes of different proportion of Ad. Table S4. Odds ratio difference between heterogeneous and autogenous samples in vignettes of different proportion of Ad. Table S5. Interaction estimation between CpG methylation and age, gender, TNM in Ad and Sc. [file 1868-7083-6-5-S1.docx]

Additional file 1: Table S1. TCGA probe information in this study

| CpG ID | Chrosome | MapInfo | Strand | Accession | Distance to TSS | CpG Island |
| --- | --- | --- | --- | --- | --- | --- |
| cg01240931 | Chr5 | 112101942 | + | NM_000038.3 | -459 | FALSE |
| cg15020645 | Chr5 | 112101668 | + | NM_000038.3 | -185 | FALSE |
| cg16970232 | Chr5 | 112101332 | + | NT_034772.5 | 151 | TRUE |
| cg20311501 | Chr5 | 112101401 | + | NT_034772.5 | 82 | TRUE |
| cg21634602 | Chr5 | 112101469 | + | NM_000038.3 | 14 | TRUE |
| cg24332422 | Chr5 | 112101585 | + | NM_000038.3 | -102 | TRUE |

CpG ID is from illumina microarray platform. MapInfo is the location of the CpG site based on **Human Genome Source Version 36.1**. Distance was negative when the site locates in the promoter region of the APC gene. TSS represent transcription start site. TSS coordination is Chr5: 112101483.

Table S2. Three kinds of primers of present 17 studies

| Study | Forward | Reverse | Type |
| --- | --- | --- | --- |
| Pan et al (2009,China) | ACTGCCATCAACTTCCTTGCTTGCT* | GACATGTGGCTGTATTGGTGCAGCCCG | chr5:112073311-112073571 |
| Zhang et al (2011,China)^b^ | CACTGCGGAGTGCGGGTC | CCGTCGGGAGCCCGCCGA | chr5:112073421+112073518 |
| Virmani et al (2001, USA) | CACTGCGGAGTGCGGGTC | CCGTCGGGAGCCCGCCGA | chr5:112073421+112073518 |
| Kim et al (2007, Korea) | CACTGCGGAGTGCGGGTC | CCGTCGGGAGCCCGCCGA | chr5:112073421+112073518 |
| Lin et al (2009, China) | CACTGCGGAGTGCGGGTC | CCGTCGGGAGCCCGCCGA | chr5:112073421+112073518 |
| Shivapurkar et al (2007, USA) | CACTGCGGAGTGCGGGTC | CCGTCGGGAGCCCGCCGA | chr5:112073421+112073518 |
| Suzuki et al (2006, Japan) | CACTGCGGAGTGCGGGTC | CCGTCGGGAGCCCGCCGA | chr5:112073421+112073518 |
| Zhang et al (2011, China)^b^ | CACTGCGGAGTGCGGGTC | CCGTCGGGAGCCCGCCGA | chr5:112073421+112073518 |
| Rykova et al (2004, Russia) | CACTGCGGAGTGCGGGTC | CCGTCGGGAGCCCGCCGA | chr5:112073421+112073518 |
| **Begum et al (2011, USA)** | **GGACCAGGGCGCTCCCCAT** | **GTGTGGGCGCACGTGACCGACATGTGG** | **chr5:112101379+112101452** |
| **Usadel et al (2002, USA)** | **GGACCAGGGCGCTCCCCAT** | **GTGTGGGCGCACGTGACCGACATGTGG** | **chr5:112101379+112101452** |
| **Jin et al (2009, Japan)** | **GGACCAGGGCGCTCCCCAT** | **GTGTGGGCGCACGTGACCGACATGTGG** | **chr5:112101379+112101452** |
| **Feng et al (2008, USA)** | **GGACCAGGGCGCTCCCCAT** | **GTGTGGGCGCACGTGACCGACATGTGG** | **chr5:112101379+112101452** |
| **Brabender et al (2001, USA)** | **GGACCAGGGCGCTCCCCAT** | **GTGTGGGCGCACGTGACCGACATGTGG** | **chr5:112101379+112101452** |
| **Vallbohmer et al (2006, USA)** | **GGACCAGGGCGCTCCCCAT** | **GTGTGGGCGCACGTGACCGACATGTGG** | **chr5:112101379+112101452** |

The primers of the study of Wang et al (2008, China), Topaloglu et al (2004, USA) and Yanagawa et al (2003, Japan) can't be located.

Table S3. The fluctuation of odds ratio in vignettes of different proportion of Ad

| Ad : Sc | 2:1 | | 4:3 | | 3:4 | | 1:2 | |
| --- | --- | --- | --- | --- | --- | --- | --- | --- |
| CpG Site | OR | P-values | ORs | P-values | ORs | P-value | OR | P-value |
| cg15020645 | 7.8-173.4 | 0.001 | 11.64-56.37 | <10^-4^ | 4.86-25.90 | 0.02 | 3.84-16.44 | 0.572 |
| cg16970232 | 17.2-186.4 | **<10^-4^** | 18.99-72.49 | **<10^-4^** | 14.24-50.49 | **<10^-4^** | 8.9-31.73 | **<10^-4^** |
| cg20311501 | 6.4-76.6 | **<10^-4^** | 8.09-36.23 | **<10^-4^** | 5.84-20.38 | **<10^-4^** | 3.49-13.58 | **0.001** |
| cg21634602 | 3.3-31.14 | 0.014 | 3.72-12.78 | <10^-4^ | 2.78-8.30 | 0.022 | 1.74-6.33 | 0.582 |
| cg24332422 | 4.6-203.3 | 0.006 | 9.17-48.01 | <10^-4^ | 5.47-22.77 | 0.012 | 2.79-12.21 | 0.546 |

10000 random resampling were conducted to simulate the bias of the odds ratios of each CpG site in vignettes of different proportion of Ad. P-value was defined as the probability of each simulation whose p-value>0.05. Sample sizes were 400:300,300:300,150:300,400:200 in above four vignettes, respectively

Table S4. Odds ratio difference between heterogeneous and autogenous samples in vignettes of different proportion of Ad

| Ad : Sc | 2:1 | | | 4:3 | | | 3:4 | | | 1:2 | | |
| --- | --- | --- | --- | --- | --- | --- | --- | --- | --- | --- | --- | --- |
| CpG Site | OR_a_ | OR_h_ | p-value | OR_a_ | OR_h_ | p-value | OR_a_ | OR_h_ | P-value | OR_a_ | OR_h_ | P-value |
| cg15020645 | 572.4 | 43.36 | **<10^-4^** | 264.6 | 24.41 | **<10^-4^** | 0.27 | 3.1 | **<10^-4^** | 3.26 | 12.6 | **<10^-4^** |
| **cg16970232** | 149.2 | 59.40 | **<10^-4^** | 83.3 | 37.84 | **<10^-4^** | 14.1 | 16.3 | **<10^-4^** | 11.42 | 21.5 | **<10^-4^** |
| cg20311501 | 44.6 | 24.88 | **<10^-4^** | 24.4 | 17.61 | **<10^-4^** | 20.3 | 25.5 | **<10^-4^** | 3.31 | 10.1 | **<10^-4^** |
| cg21634602 | 23.8 | 9.80 | **<10^-4^** | 12.6 | 7.53 | **<10^-4^** | 6.1 | 12.6 | **<10^-4^** | 1.88 | 4.8 | **<10^-4^** |
| cg24332422 | 192.7 | 42.02 | **<10^-4^** | 64.7 | 24.46 | **<10^-4^** | 3.3 | 5.84 | **<10^-4^** | 2.42 | 11.1 | **<10^-4^** |

10000 random resampling were conducted to simulate the bias of the odds ratios of each CpG site. P-value was estimated by t-test. Sample sizes were 300:400,400:300, 560:280, 200:400 in above four vignettes, respectively. OR_a_, OR_h_ represent odds ratio of each CpG site based on autogenous and heterogeneous samples.

Table S5. Interaction estimation between CpG methylation and age, gender, TNM in Ad and Sc

| CpG Site | LUAD | | | | | | LUSC | | | | | |
| --- | --- | --- | --- | --- | --- | --- | --- | --- | --- | --- | --- | --- |
|  | Age | | Gender | | TNM | | Age | | Gender | | TNM | |
|  | β | pvalue | β | pvalue | β | pvalue | β | pvalue | β | pvalue | β | pvalue |
| cg15020645 | 0.06 | 0.61 | 0.90 | 0.69 | -14.24 | 0.83 | 0.02 | 0.91 | -1.36 | 0.70 | -0.47 | 0.46 |
| cg16970232 | 0.07 | 0.47 | -0.26 | 0.90 | -14.05 | 0.98 | 0.04 | 0.71 | -1.48 | 0.56 | 0.32 | 0.50 |
| cg20311501 | 0.04 | 0.63 | -0.39 | 0.83 | -14.07 | 0.96 | 0.03 | 0.77 | -0.56 | 0.77 | 0.37 | 0.35 |
| cg21634602 | 0.04 | 0.59 | 0.34 | 0.82 | -13.92 | 0.83 | 0.04 | 0.60 | -0.17 | 0.92 | 0.36 | 0.29 |
| cg24332422 | 0.07 | 0.56 | 0.66 | 0.79 | -14.29 | 0.89 | 0.01 | 0.93 | -2.96 | 0.28 | -0.09 | 0.86 |

Betas were based on logistic regression with the function: Y~CpG +age*CpG, or Y~CpG + gender*CpG, or Y~CpG + TNM*CpG, respectively.
