# Supplementary material for: Quantitative assessment of the diagnostic role of APC promoter methylation in non-small cell lung cancer
Source: Clin Epigenetics. 2014 Mar 24;6(1):5. doi: 10.1186/1868-7083-6-5 (PMC3997934; doi:10.1186/1868-7083-6-5)

**Additional file 2** Figure S1. Funnel plot to diagnosis of the publication bias. Figure S2. Combined estimates for the association between APC methylation and NSCLC after trim-fill treatment. Figure S3. Sensitivity analyses of the overall effect by omitting a single study.

Figure S1. Funnel plot to diagnosis of the publication bias



Figure S2. Combined estimates for the association between APC methylation and NSCLC after trim-fill treatment.


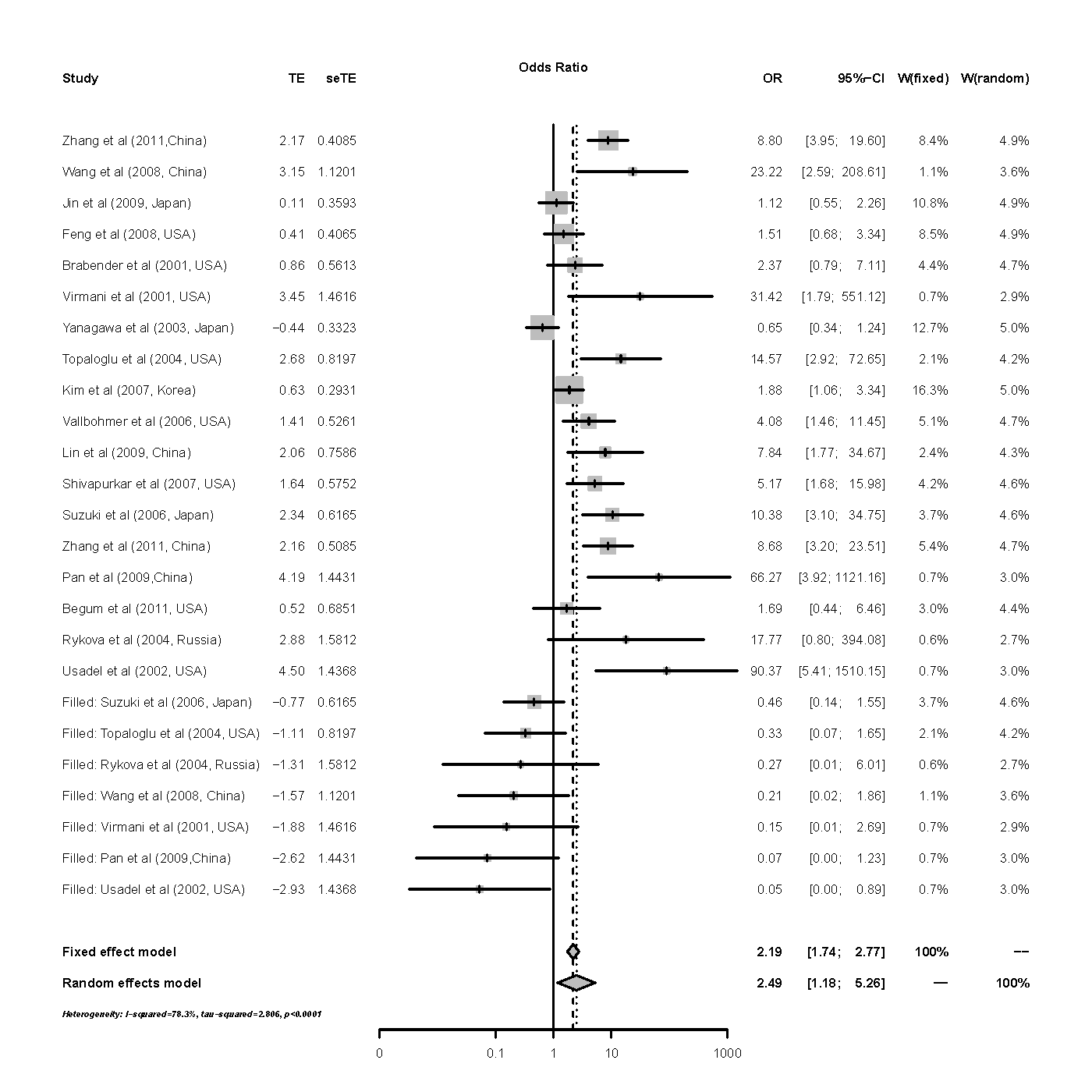


Figure S3. Sensitivity analyses of the overall effect by omitting a single study.


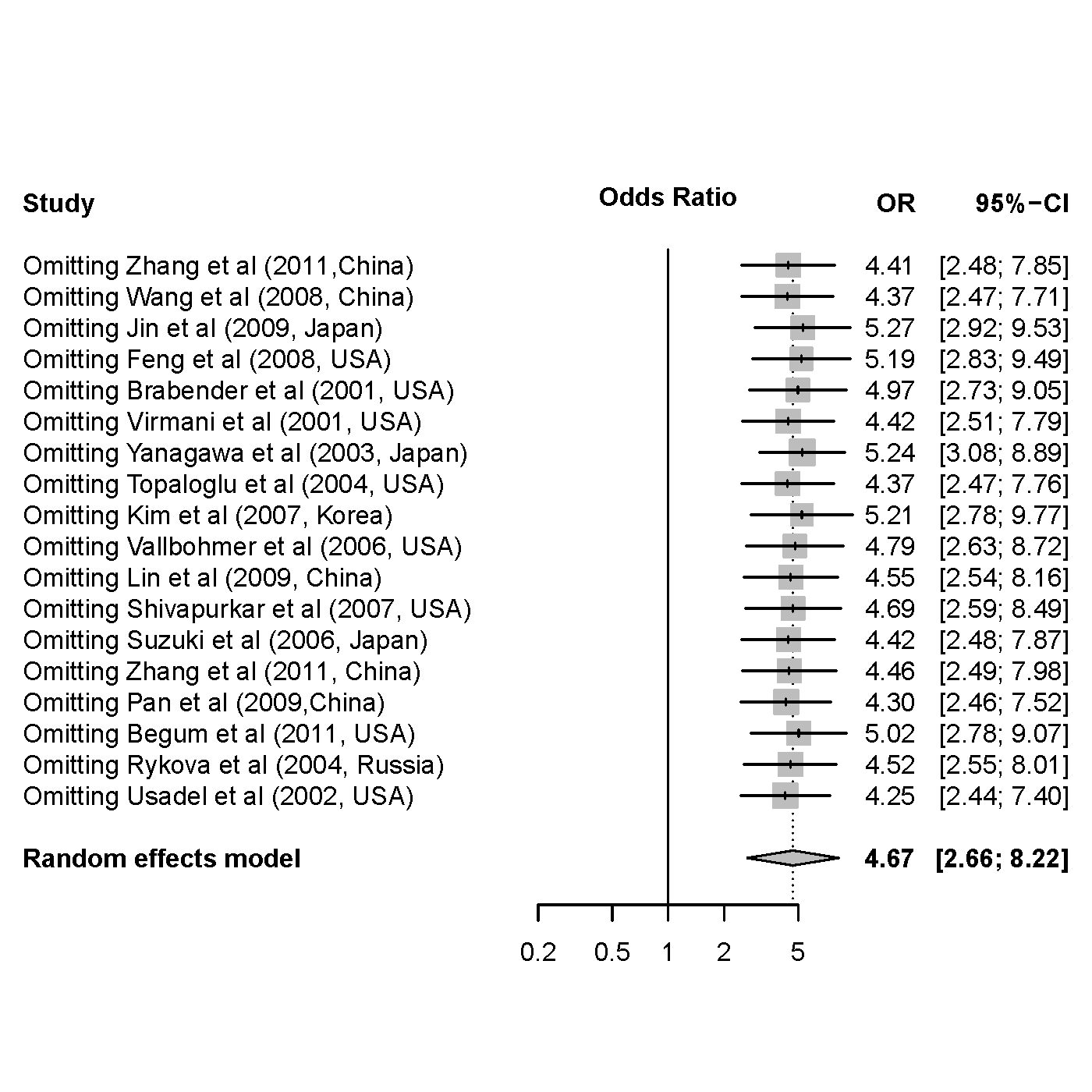

Supplement: Additional file 2: Figure S1 — Funnel plot to diagnosis of the publication bias. Figure S2. Combined estimates for the association between APC methylation and NSCLC after trim-fill treatment. Figure S3. Sensitivity analyses of the overall effect by omitting a single study. [file 1868-7083-6-5-S2.docx]
